# Oral Lesions and Lymphoproliferative Disorders

**DOI:** 10.1155/2010/202305

**Published:** 2010-09-01

**Authors:** P. Castellarin, G. Pozzato, G. Tirelli, R. Di Lenarda, M. Biasotto

**Affiliations:** ^1^Department of Dental Science, University of Trieste, 34127 Trieste, Italy; ^2^Department of Haematology, University of Trieste, 34142 Trieste, Italy; ^3^Department of Otorhinolaryngology, Head and Neck Surgery, University of Trieste, 34127 Trieste, Italy

## Abstract

Lymphoproliferative disorders are heterogeneous malignancy characterized by the expansion of a lymphoid clone more or less differentiated. At the level of the oral cavity, the lymphoproliferative disorder can occur in various ways, most commonly as lymphoid lesions with extranodal externalization, but sometimes, oral lesions may represent a localization of a disease spread. With regard to the primary localizations of lymphoproliferative disorders, a careful examination of the head and neck, oral, and oropharyngeal area is necessary in order to identify suspicious lesions, and their early detection results in a better prognosis for the patient. Numerous complications have been described and frequently found at oral level, due to pathology or different therapeutic strategies. These complications require precise diagnosis and measures to oral health care. In all this, oral pathologists, as well as dental practitioners, have a central role in the treatment and long-term monitoring of these patients.

## 1. Introduction

Under the name of lymphoproliferative disorders various disease patterns are included which are characterized by the expansion of a lymphoid clone more or less differentiated. The application in recent times, of immunological methods for determining the phenotype of many cell components, together with the acquisitions of cytogenetic and molecular biology, as well as clinical behavior, have helped to relatively define a wide range of diseases that may present a heterogeneous clinical and morphological picture. In fact, the last classification of lymphoproliferative disorders lists 40 types of lymphoproliferative syndromes to immunophenotype B and 23 to immunophenotype T [1]. At the level of the oral cavity, the lymphoproliferative disorder can occur in various ways, most commonly as lymphoid lesions with extranodal externalization, but sometimes, oral lesions may represent a localization of a disease spread [2]. Under the key research that sees lymphoproliferative disorders associated with injury or events at the oral cavity, the present paper proposes a comprehensive classification as listed in Table 1 and deeply described below.

## 2. Classification and Related Aspects of the Oral Pathologies Associated with Lymphoproliferative Disorders

### 2.1. Group 1: Primary Oral Lymphoproliferative Disorders Limited to the Oral Cavity that Will not Invade Other Body Districts

**Figure 1:**
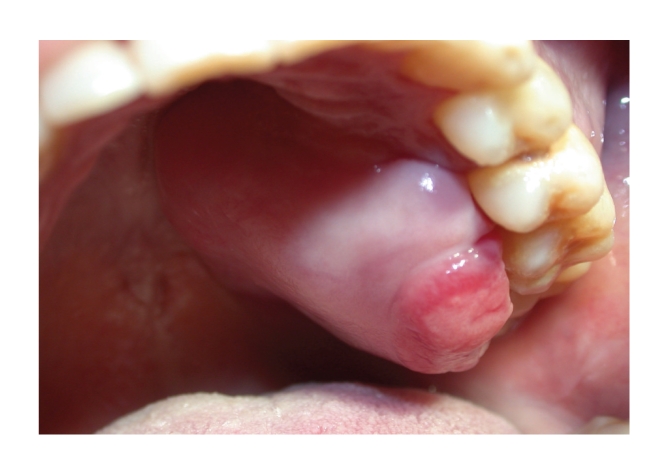
Primary extranodal non-Hodgkin lymphoma: painless mass involving the left palate.

Primary extranodal involvement can be seen in 10% to 35% of cases of *non-Hodgkin lymphomas.* These locations include the gastrointestinal tract, skin, testicles, kidneys, and bones [3, 4]; the NHL of the central nervous system account for 1% of cases [5, 6]. Although the oral involvement of NHL is rare, they are the second most common oral malignant disease after oral squamous cell carcinoma [7, 8], constituting of 2.2% of all malignancies of the head-neck, 3.5% of intraoral malignancies, 5% of tumors of the salivary glands, and 2.5% of all cases of NHL [8]. Although every other site may be affected, Ring Waldayer is the most commonly involved [9]. The WHO system classifies NHL as indolent, aggressive, and highly aggressive. Indolent lymphoma accounts for 40% af all NHL with the most common type being follicular lymphoma; aggressive lymphoma accounts for approximately 50% of cases and include diffuse large B-cell lymphoma and T-cell natural killer (NK) cell lymphoma; highly aggressive lymphoma includes EBV-associated Burkitt lymphoma and lymphoblastic lymphoma [10]. Even though each type of NHL can occur at the level of oral cavity, the most common types are large-cell lymphoma and lymphoma of small lymphocytes lymphoma; mucosa-associated lymphoid tissue (MALT) lymphoma, follicular lymphoma, Burkitt's lymphoma, and immunocytoma, immunoblastic lymphoma are also reported [8, 11, 12]. The most common signs and symptoms with which the NHL will have intraoral include tissue swelling, tooth loss, and paresthesia. The radiographic measurements can highlight widespread osteolysis with loss of lamina dura. Oral lesions may appear as exophytic neoplasms, erythematous, asymptomatic, often with superficial ulcerations secondary to chronic trauma [11] (Figure 1).

*Hodgkin's disease* is a form of malignancy characterized by proliferation of neoplastic cells (Hodgkin and Reed-Sternberg cells) associated with a polymorphic cellular component (lymphocytes, histiocytes, eosinophils, neutrophils, and plasma cells), considered to be reactive [13]. From its initial description until the 1990s, the nature and lineage of the Reed-Sternberg cell and the inflammatory infiltrate that compromises Hodgkin disease were debated. The application of PCR technique revealed that Hodgkin and Reed-Sternberg cells were clonal B cells in 98% of the patients, leading to the change of the designation of Hodgkin disease into *Hodgkin lymphoma* (LH) in the WHO classification of lymphoid neoplasms [1]. Primary oral and oropharyngeal lesions are rare [14]. Since the Waldayer ring is commonly involved by NHL that sometimes shows cells of Reed-Sternberg cell-like, the diagnosis of LH is difficult, especially when there are only small biopsy specimens [15, 16]. The most common site involved is the ring of Waldayer followed by the lips [17], tongue base [18, 19], buccal mucosa [20, 21], and parotid gland [22, 23].

The oral manifestations by plasma cell tumors can occur in three different ways: as a consequence of the local manifestation of multiple myeloma, bone plasmacytoma as solitary, or as extramedullary plasmacytoma [24]. The primary manifestations of plasma cell neoplasms at the oral level are represented by solitary and extramedullary plasmacytoma. 

**Figure 2:**
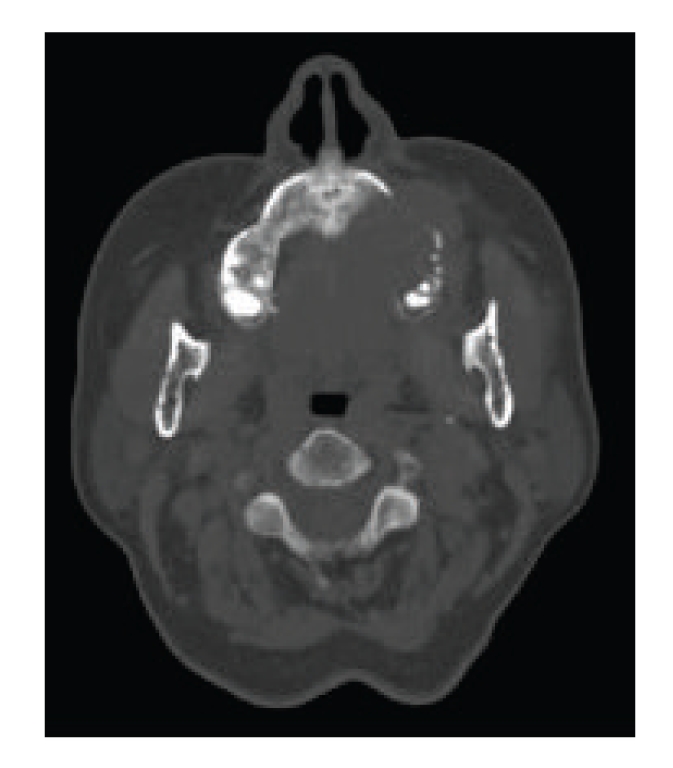
Plasmacytoma: axial noncontrast CT image at the level of left maxillary sinus demonstrates an expansile, destructive intraosseous lesion involving the left maxillary sinus.

The *Plasmacytoma* of bone can be considered a localized solitary myeloma (Figure 2). Solitary bone plasmacytoma is a malignant monoclonal gammopathy [25, 26]; it is a plasma cell cancer that occurs as a single osteolytic lesion without plasmacytosis of the bone marrow and that is capable of secreting monoclonal M protein [27, 28]. This disease accounts for 10% of all plasma cell tumors and can strike [25, 27, 28], although rarely, in the oral cavity, showing a predilection for the mandibular retromolar area [29]; its radiological appearance may have one of two patterns, as either an oval-shaped lytic image with destruction of the cortical bone, or as a hyperinsufflating lesion showing a convex bicortical bone [30].

Instead, the extramedullary plasmacytoma is a plasma cell tumor located separately from the bone marrow [31]; it is found in all parts of the body where this lymphoid tissue [32–38], in 90% of the extramedullary plasmacytoma, is present in the head and neck. Clinically [39–41], the plasmacytoma extraosseous is present as a sessile or pedunculated exophytic neoformation, circumscribed or infiltrative, ranging in color from red-purple to gray or yellow-white [42–50].

### 2.2. Groups 2: Primary Oral Lymphoproliferative Disorders that May Eventually Invade Other Body Districts

The oral manifestations of multiple myeloma (MM), a disease characterized by the proliferation and accumulation in the bone marrow of a clone of plasma cells to produce a homogeneous monoclonal protein [13, 30, 51, 52], are extensively reported in the literature; the oral manifestations are the initial sign of submission in 12% to 15% of cases of MM [53–55]. The oral features include facial, oral, and dental pain, numbness and paresthesia, swelling, soft tissue neoplasms [56], tooth mobility, bleeding, and deposit of amyloid substance especially on the tongue [57–60]. Other examples of oral lesions as first manifestation of lymphoproliferative disorders may be the infiltration of the oral mucosa by a *B-cell chronic lymphocytic leukemia* [61], the *lymphomatous papulosis*, a condition mucocutaneous applicant, self-limited, characterized by papular eruptions [62], and *Mycosis fungoides*, T-cell cutaneous lymphoma in which the involvement of the oral cavity is a rare event but well documented [63, 64].

### 2.3. Group 3: Primary Systemic Lymphoproliferative Disorders That May Eventually Invade Oral Cavity

In addition to entering into this category, the previously treated lymphoproliferative disorders, where the diagnosis of oral lymphoid malignancy was subsequent to an indication of their early sign, mentioned in this regard a clinical-pathological entity of which we have evidence in the literature for over a century of mycosis fungoides (MF), chronic lymphoproliferative disorder with predominantly cutaneous involvement characterized by the proliferation of T lymphocytes that in advanced stages of the disease can accumulate also in lymphoid organs, bone marrow, and peripheral blood (Sezary Syndrome) [13]. Involvement in oral MF is an occasional finding observed from 7.4% to 18% of patients undergoing necropsy [65, 66]. Despite these findings relatively frequent mucosal involvement in vivo is a rare event; there is no predisposition to sex; age of onset varies from 36 to 81 years, with an average of 61 years. Oral sites most frequently involved are the tongue, palate, gingiva, buccal mucosa, lips, and oropharynx. In almost all patients, the lesions skin prior to the mucosa over a period of time ranging from 7 months to 40 years [67].

### 2.4. Groups 4: Primary Oral Lesions (Excluding Lymphoproliferative Disorders) That Are Associated with Systemic Lymphoproliferative Disorders, That Is, Paraneoplastic Pemphigus

Here is a list of non-specific signs and symptoms present in association with lymphoproliferative disorders of the oral cavity: lymphadenopathy, trismus, erythema, epiphora, pain, swelling, facial asymmetry or swelling of buccal mucosa, sinusitis, increased lacrimation and abscesses of the lacrimal sac, diplopia, nasal obstructions, sepsis, fever, runny nose, prosthetic instability, headache, and paresthesia idiopathic epistaxis. Suspicion of malignancy usually develops only after these inflammatory symptoms have not responded to conventional treatment protocols, upon which more accurate evaluations are required. Although oral lymphomas are extremely rare [68, 69], they can occur earlier and be placed in the differential diagnosis with non-specific inflammatory processes. Moreover, the early recognition of these subtle cancers can decrease their morbidity [70].

**Figure 3:**
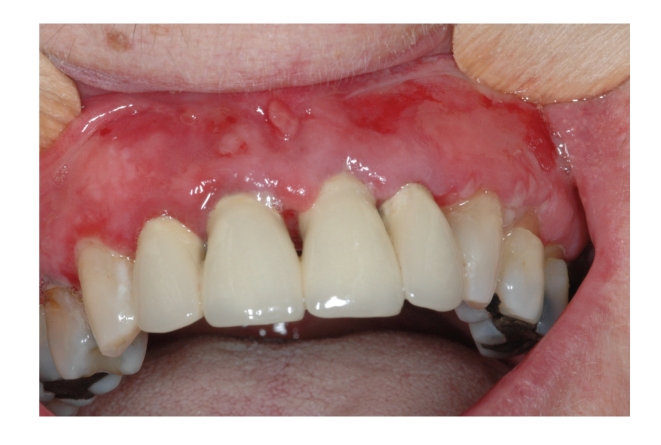
Paraneoplastic pemphigus: multiple erosions affecting the marginal gingival.

*Paraneoplastic pemphigus* (PNP), or paraneoplastic autoimmune multiorgan syndrome is a rare autoimmune vesiculobullous disease first described by Anhalt et al. in 1990 in patients with occult malignancies [71, 72]. The PNP may have mucocutaneous and systemic manifestations. Erosive eye lesions, sinuses, oral cavity, the gastrointestinal system, and respiratory and genital epithelium could affect them. Clinically, lesions may occur as polymorphic, such as pemphigus, bullous pemphigoid, erythema multiforme, the graft-versus-host disease, and lichen planus [73–75] (Figure 3).

*Dermatomyositis* (DM) is a rare inflammatory microangiopathic disease that affects skeletal muscles, with clinical externalization as characteristics mucocutaneous manifestations.

Oral lesions in paraneoplastic DM (leucoerythroplasia and ulcerative lesions) have rarely been described in the literature [76–79]. The DM is totally resolved if the underlying disease is treated and the resurgence of DM expresses relapse of malignancy [76, 79].

In the literature, there are reports of a single case of *multiple myeloma* with first manifestation at the oral level under the clinical aspects of lichen planus, showing extensive and irregular erosions present at buccal mucosa, labial, palatal mucosa, and ventral tongue [80].

### 2.5. Group 5: Primary Lymphoproliferative Disorders That May Eventually Invade Oral Cavity Yielding Lesions (Excluding Lymphoproliferative Disorders)

The literature contains many works that correlate the *paraneoplastic pemphigus* (PNP), a rare autoimmune vesiculobullous disease underlying a malignancy [71, 72], with typical oral lesions of lichen planus [81] or lichenoid reactions [82]. In the literature there are numerous works reported that associate *mucous membrane pemphigoid* (MMP) with malignancies, including lung cancer [83], pancreatic adenocarcinoma [84], gastric adenocarcinoma [85], and squamous cell carcinoma of the conjunctiva [86] there are only two cases reported of MMP with oral manifestations associated to lymphoproliferative disorders: a B-cell lymphoma [87] and chronic lymphocytic leukemia [88]. It has been described in the literature that large amount of cases have connection between *bullous pemphigoid* (BP) and lymphoproliferative disorders such as chronic lymphocytic leukemia [89–91] and lymphoblastic lymphoma [92].

*Necrotizing oral processes*, although rare in the general population, can rapidly evolve in devastating stages in immunocompromised patients [93–96] and are often associated with periodontal disease. These are patients with lymphoproliferative disorders such as acute lymphoblastic leukemia, which developed necrotic processes at the level of the oral cavity not associated with typical ulcer-necrotizing periodontal diseases, but by bacteria in the oral cavity unusually found as *Pseudomonas aeruginosa*. Patients with impaired lymphocyte function or reduced counts of neutrophils, due to lymphoproliferative disorders, have led to the acquisition of new infections and/or exacerbation or reactivation of latent infection (periapical periodontitis and herpes simplex). In many cases the clinical presentation of oral infections may be atypical when compared to those normally seen in healthy patients.

The origin of *odontogenous infection* of the pulp and periodontal is frequently observed in patients with lymphoproliferative disorders and should be suspected when orofacial pain, extensive dental restorations, and dental caries and periapical radiopacity occur [97, 98].

Oropharyngeal candidiasis is the most common *fungal infection* in cancer patients [99–101]. The candidiasis may present as pseudomembranous candidiasis, erythematous, hyperplastic, or angular cheilitis. The symptoms include general discomfort, dysgeusia, xerostomia, and mouth burn. Deep fungal infections should be suspected in patients with solitary ulcer that does not retreat. This group includes infections such as aspergillosis, histoplasmosis, and that by *Zygomycetes*, and the treatment requires aggressive treatment with intravenous azoles, amphotericin B, and echinocandins [102]. 

Primary infection or reactivation of herpes viruses is common in patients with lymphoproliferative disorders especially during the advanced stages of the disease. Infection with herpes simplex is most common in these patients; the reactivation of the *Varicella-Zoster *virus (VZV) is less common [103]. *Epstein-Barr *virus infection (EBV) is associated with oral hairy leucoplakia, a very rare whitish lesion that occurs on the lingual lateral margins in myelosuppressive patients [104, 105]. Infection by *Cytomegalovirus* (CMV) can occur on any intraoral mucosal surface in the form of an ulcer-free character of specificity, which persists for weeks or months.

**Figure 4:**
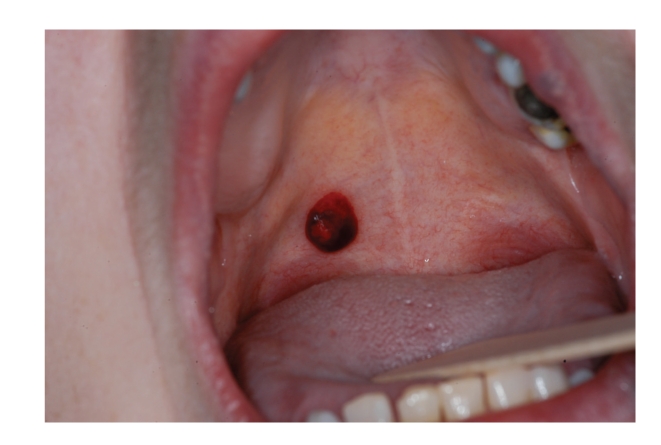
Thrombocytopenia: traumatic hematoma of the soft palate.

Because of *thrombocytopenia* in the course of lymphoproliferative disorders, intraoral bleeding is commonly observed, which is present with petechiae, bruising, and occasionally with the formation of hematomas (Figure 4).

### 2.6. Group 6: Oral Complications Due to Systemic Treatment for Lymphoproliferative Disorders (i.e., Chemotherapy, Radiotherapy, Autologous, or Allogeneic Bone Marrow Transplant)

**Chemotherapy and Radiotherapy**

In addition to what is included in this paper the oral infectious complications (bacterial, fungal, viral), previously treated on immunosuppression induced by lymphoproliferative disorder, and here remembered as a possible consequence of drug-induced immunodeficiency, we will consider the common characteristic found in the course of oncohaematology treatment regimens. Patients with lymphoproliferative disorders who are treated with high doses of radiotherapy at the head and neck level, associated with aggressive chemotherapy regimens, are at greatest risk of developing oropharyngeal mucositis. Xerostomia is often present in patients with lymphoproliferative disorders treated with radiotherapy in the head and neck region due to damage of the major salivary glands, which are still included in the irradiation of tumors. As another long-term complication of radiation therapy for head and neck lymphomas in patients who underwent radiotherapy, we noticed the osteoradionecrotic maxillary bone as a result of permanent damage of the capillary bed of bone and osteocytes caused by radiation.

**Autologous Transplantation**

Oral complications are observed in 85% of cases of patients after autologous bone marrow transplantation (ABMT). These complications are due to the effects derived from antiblastic chemotherapy and radiotherapy. Infections occur during the period of severe marrow aplasia and may be secondary to the conditions of the oral cavity before the transplant. Patients treated for lymphoproliferative disorders are at increased risk of relapse of the primary disease so as to develop subsequent tumors both hematologic [106] and nonhematologic in this regard we consider the oral squamous cell carcinoma as a second primary solid tumor after allogeneic transplant [107].

A variety of circulating autoantibodies can be found from 20% to 61% of patients undergoing autologous bone marrow transplant [108–111], and a number of these patients may demonstrate autoantibody-mediated diseases such as myasthenia gravis and autoimmune cytopenias [112] and some cases of oral diseases, such as oral vesiculobullous (pemphigus and pemphigoid) [113–116].

**Figure 5:**
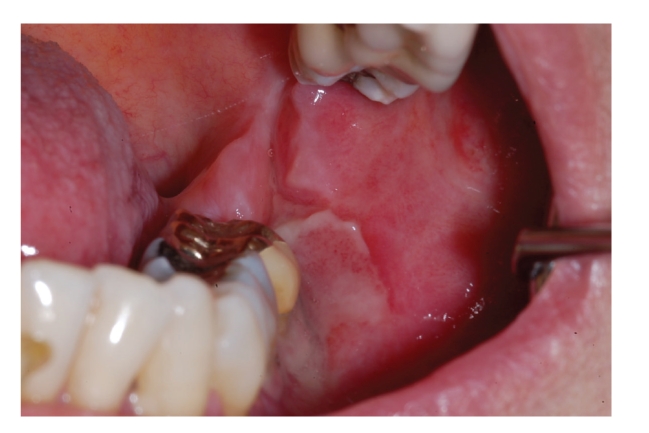
Oral cGVHD: ulceration of the buccal mucosa.

The Graft-versus-host disease (GVHD) is a multisystem immunologic reaction resulting from the action of immunocompetent cells transplanted from a donor to an immunodeficient host [117]. The recipients of *allogeneic hematopoietic cell transplantation* (allo-HTC) are at greater risk of developing the disease from acute and chronic graft versus host. The likelihood of developing GVHD depends on the type of conditioning regimen (ablative vs. totally reduced intensity regimens). GVHD in oral lesions, although infrequent, can be observed, and rarely require specific treatment. Chronic GVHD typically develops after the hundredth day, with an incidence ranging from 25% to 40% after allo-HCT [107, 117] (Figure 5). The mouth is one of the locations most commonly affected by events such as lichenoid mucositis (including ulcers), pain, odynophagia, dysgeusia, hypofunction of the salivary glands, caries decayed teeth, and rarely sclerotic processes that lead to a hypomobility and a reduction in functionality [10, 118].

**Supportive Therapy**

**Figure 6:**
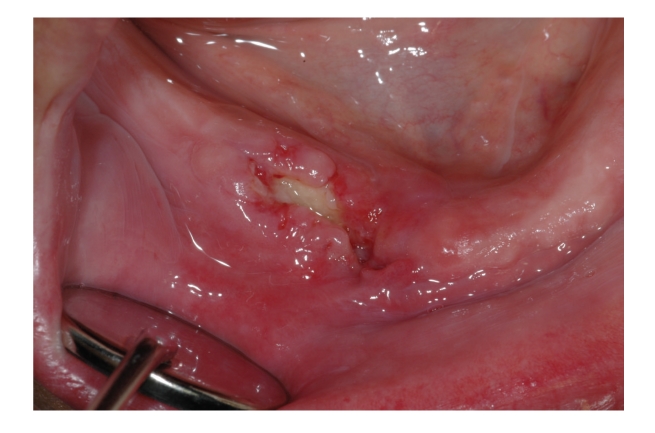
Bisphosphonates-related osteonecrosis of the jaw. Spontaneous necrotic bone exposure.

Bisphosphonates are drugs used in combination with chemotherapy in the treatment of hypercalcemia secondary to malignancy, lytic bone metastases and multiple myeloma [119–121]. Starting from 2003, an increasing number of cases that describe the correlation between osteonecrosis of the jaw (ONJ) and administration of intravenous bisphosphonates were published in the literature [122–131] (Figure 6).

### 2.7. Group 7: AIDS-Related Lymphoproliferative Disorders of the Oral Cavity

Approximately 10% of all cases of non-Hodgkin lymphomas in the United States and Europe are AIDS related [132–134]. Typically, AIDS-related NHL has a predilection for extranodal sites and appears in the head and neck, in 50-60% of cases [135–144]; they have an aggressive course and a poor prognosis [145–147]. In histopathology these lymphomas are B-cells derived and characterized by widespread growth, pleomorphism, high-grade morphology, frequent mitotic figures, and increased tendency to develop into necrosis [148–153]. Lymphomas of head and neck may involve the gingiva, palate, tongue, tonsils, nasopharynx, orbits, parotid glands, jaw breasts, soft tissues, and hypopharynx. These tumors are associated with severe immunosuppression and a poor response to chemotherapy.

## 3. Conclusions

Lymphoid neoplasms are heterogeneous malignant disorders of the lymphatic system characterized by uncontrolled proliferation of lymphoid cells or their precursors. With regard to the primary localizations of lymphoproliferative disorders, a careful examination of the head and neck, oral, and oropharyngeal area is necessary in order to identify suspicious lesions and their early detection. An early diagnosis may allow treatment at early stages of the disease, resulting in a better prognosis for the patient. Numerous complications have been described and frequently found at oral level, due to pathology or different therapeutic strategies. These complications require a precise diagnosis and measures to oral health care. Furthermore, the evaluation of patients after treatment for lymphoma is essential to prevent relapse and to recognize early any eventual secondary localizations.

In all this, oral pathologists, as well as dental practitioners, have a central role in the treatment and long term monitoring of these patients, having the opportunity and the duty to examine and make a diagnosis at the level of hard and soft tissue of the oral cavity.

## Figures and Tables

**Table 1:** Classification of the oral pathologies associated with lymphoproliferative disorders (see text for details).

Group	Primary disorder	Localization of the primary disorder	Secondary (a) or associated (b) disorder	Localization of the secondary or associated disorder
1	Lymphoproliferative disorders	Oral cavity	None	—
2	Lymphoproliferative disorders	Oral cavity	Lymphoproliferative disorders (a)	Other body districts
3	Lymphoproliferative disorders	Other body districts	Lymphoproliferative disorders (a)	Oral cavity
4	Oral lesions	Oral cavity	Lymphoproliferative disorders (b)	Other body districts
5	Lymphoproliferative disorders	Other body districts	Oral lesions (a)	Oral cavity
6	Lymphoproliferative disorders	Other body districts	Oral lesions secondary to the treatment of the primary disorder	Oral cavity
7	AIDS	—	AIDS-related lymphoproliferative disorders	Oral cavity

Oral lesions refer to all of the diseases excluding lymphoproliferative disorders.
